# Carvacrol decreases blood–brain barrier permeability post-diffuse traumatic brain injury in rats

**DOI:** 10.1038/s41598-023-40915-x

**Published:** 2023-09-04

**Authors:** Elham Abbasloo, Mohammad Khaksari, Mojgan Sanjari, Firas Kobeissy, Theresa Currier Thomas

**Affiliations:** 1 Institute of Basic and Clinical Physiology Sciences, Endocrinology and Metabolism Research Center, Kerman, Iran; 2 Institute of Neuropharmacology, Physiology Research Center, Kerman University of Medical Sciences, Kerman, Iran; 3 Department of Biochemistry and Molecular Genetics, Faculty of Medicine, American University of Beirut, Beirut, Lebanon; 4 College of Medicine-Phoenix, University of Arizona, Child Health, Phoenix, USA; 5 BARROW Neurological Institute at Phoenix Children’s Hospital, Phoenix, USA

**Keywords:** Neuroscience, Physiology

## Abstract

Previously, we showed that *Satureja Khuzestanica* Jamzad essential oil (SKEO) and its major component, carvacrol (CAR), 5-isopropyl-2-methylphenol, has anti-inflammatory, anti-apoptotic, and anti-edematous properties after experimental traumatic brain injury (TBI) in rats. CAR, predominantly found in Lamiaceae family (*Satureja* and *Oregano*), is lipophilic, allowing diffusion across the blood–brain barrier (BBB). These experiments test the hypothesis that acute treatment with CAR after TBI can attenuate oxidative stress and BBB permeability associated with CAR’s anti-edematous traits. Rats were divided into six groups and injured using Marmarou weight drop: Sham, TBI, TBI + Vehicle, TBI + CAR (100 and 200 mg/kg) and CAR200-naive treated rats. Intraperitoneal injection of vehicle or CAR was administered thirty minutes after TBI induction. 24 h post-injury, brain edema, BBB permeability, BBB-related protein levels, and oxidative capacity were measured. Data showed CAR 200 mg/kg treatment decreased brain edema and prevented BBB permeability. CAR200 decreased malondialdehyde (MDA) and reactive oxygen species (ROS) and increased superoxide dismutase (SOD) and total antioxidative capacity (T-AOC), indicating the mechanism of BBB protection is, in part, through antioxidant activity. Also, CAR 200 mg/kg treatment suppressed matrix metalloproteinase-9 (MMP-9) expression and increased ZO-1, occludin, and claudin-5 levels. These data indicate that CAR can promote antioxidant activity and decrease post-injury BBB permeability, further supporting CAR as a potential early therapeutic intervention that is inexpensive and more readily available worldwide. However, more experiments are required to determine CAR’s long-term impact on TBI pathophysiology.

## Introduction

Carvacrol (2-methyl-5-(1-methyl ethyl)-phenol) (CAR) is found in oils obtained from the plants of the Lamiaceae family, such as *Thym*, *Satureja*, and *Origanum* genera, in concentrations of 85–90%^1,2^. Because of its low molecular mass and lipophilic characteristics, this molecule may easily pass across the blood–brain barrier (BBB)^3^. CAR is generally considered a safe food additive that can be added directly to human food^4,5^ and possesses various beneficial effects in vitro and in vivo, including antioxidant, anticancer, antibacterial, antifungal, anti-inflammatory, and hepatoprotective properties^6–9^. Recent studies have shown that CAR exerts its neuroprotective effects in brain disorders by inhibiting reactive oxygen species (ROS) production and antioxidant properties^10,11^.

Traumatic brain injury (TBI) is divided into two phases of pathophysiological damage: primary (e.g., brain contusion, diffuse axonal injury, and hemorrhages of parenchyma or subarachnoid region) and secondary (e.g., BBB disruption, edema, herniation, ischemia, and infarction)^12^. Theoretically, prevention or inhibition of early secondary injury signaling cascades will attenuate persisting pathophysiology and promote improved long-term outcomes. The BBB selectively restricts the paracellular diffusion of compounds from the blood to the brain through specialized endothelial cells connected by tight junctions. Tight junctions consist of scaffolding proteins, like zonula occludens (ZO), occludins and claudin-5, that are responsible for the structural integrity of the BBB^13,14^. Astrocyte end-feet and microglial processes interact with the brain endothelium, forming the gliovascular unit responsible for maintaining cerebral homeostasis and optimal neuronal activity^15^.

**Figure 1:**
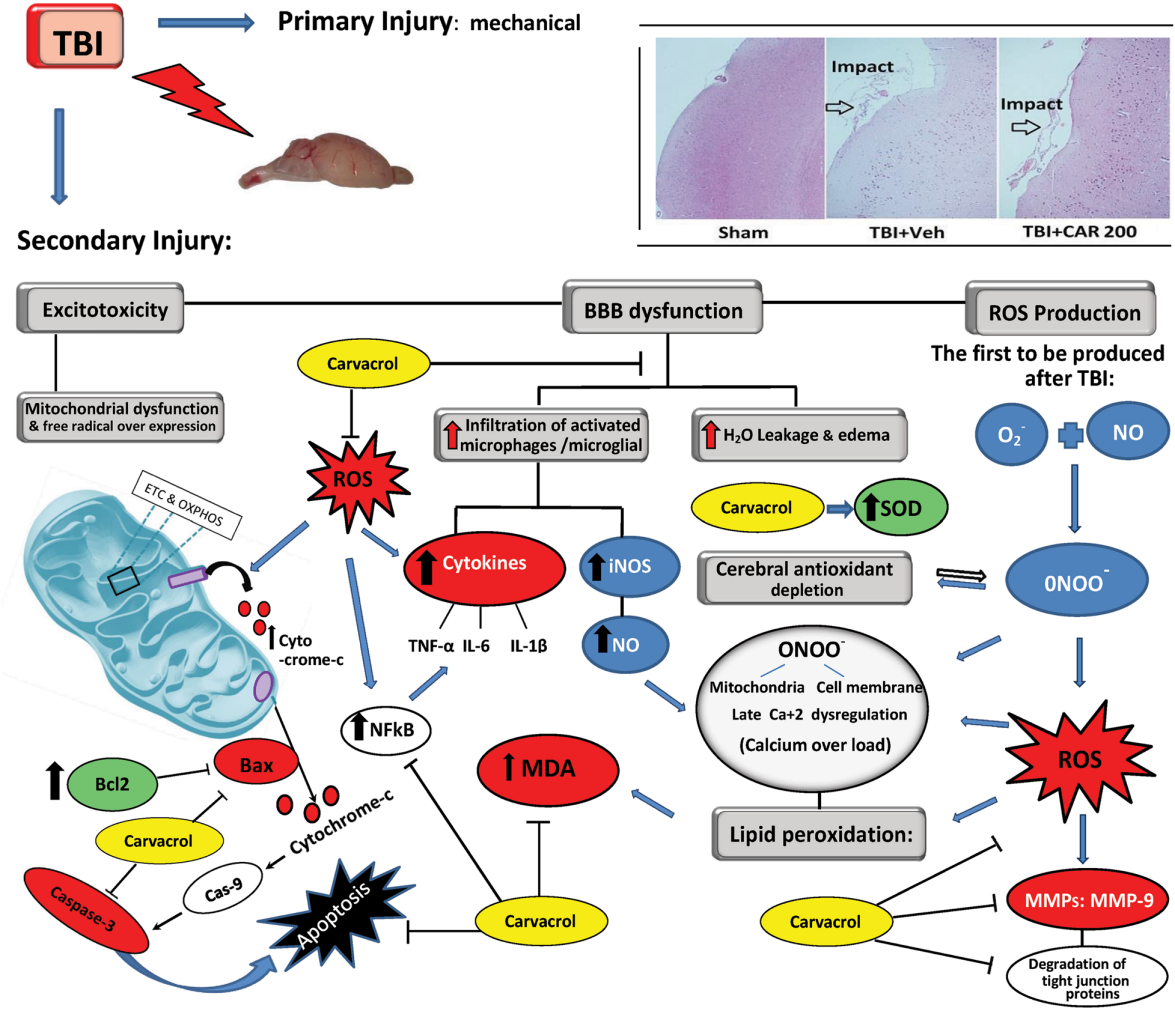
Schematic representation of the biochemical and molecular processes characterizing the TBI-mediated secondary damage. TBI induces excitotoxicity, resulting from excessive glutamate release, along with alteration of the blood–brain barrier (BBB) permeability, Malfunctioning of the mitochondrial, and free radical overexpression. TBI causes increased intracellular Ca^+2^ levels resulting from dysfunction of the mitochondrial electron transport chain (ETC) and oxidative phosphorylation (OXPHOS). This would lead to an increased nitric oxide (NO) synthase and reactive oxygen species (ROS) affecting endogenous antioxidants (e.g., glutathione peroxidase, superoxide dismutase; SOD) functions leading to oxidative stress. On the other hand, BBB permeability causes vasogenic brain edema and infiltration of activated macrophages/microglia resulting in (NO) production. Taking the processes together, ${\text{O}}_{{2}}^{ \cdot - }$ + NO would lead to peroxynitrite (${\text{ONOO}}^{ \cdot - }$) generation. Peroxynitrite and ROS actively take part in lipid peroxidation, DNA damage, and protein oxidation. Oxidative stress also wreaks havoc by modulation of vascular function, triggering cell death cascades, and activating enzymes (e.g., matrix metalloprotease-9; MMP-9). Carvacrol may alleviate the aforementioned destructive mechanisms of the cell by inhibiting ROS.

In addition to axonal injury, mild-severe TBI causes mechanical depolarization and spreading depolarization, increased intracellular Ca^+2^ levels, and decreased cerebral blood flow, resulting in a global metabolic crisis. Consequently, increased nitric oxide (NO) synthase ROS activity offset the capabilities of endogenous antioxidants (e.g., glutathione peroxidase, superoxide dismutase (SOD)), leading to oxidative stress. Oxidative stress wreaks havoc by modulation of vascular function, triggering cell death cascades, activating enzymes (e.g., matrix metalloprotease-9 (MMP-9)), damaging nucleic acids, and oxidizing fatty acids, amino acids, and co-factors of cellular processes^12,16^. Along with other secondary injury cascades, oxidative stress contributes to immediate and delayed BBB permeability allowing the diffusion of blood-borne molecules into the extracellular matrix of the brain, which further promotes oxidative and inflammatory states that lead to excessive MMP-9 activity^17,18^ (Fig. 1).

**Figure 2:**
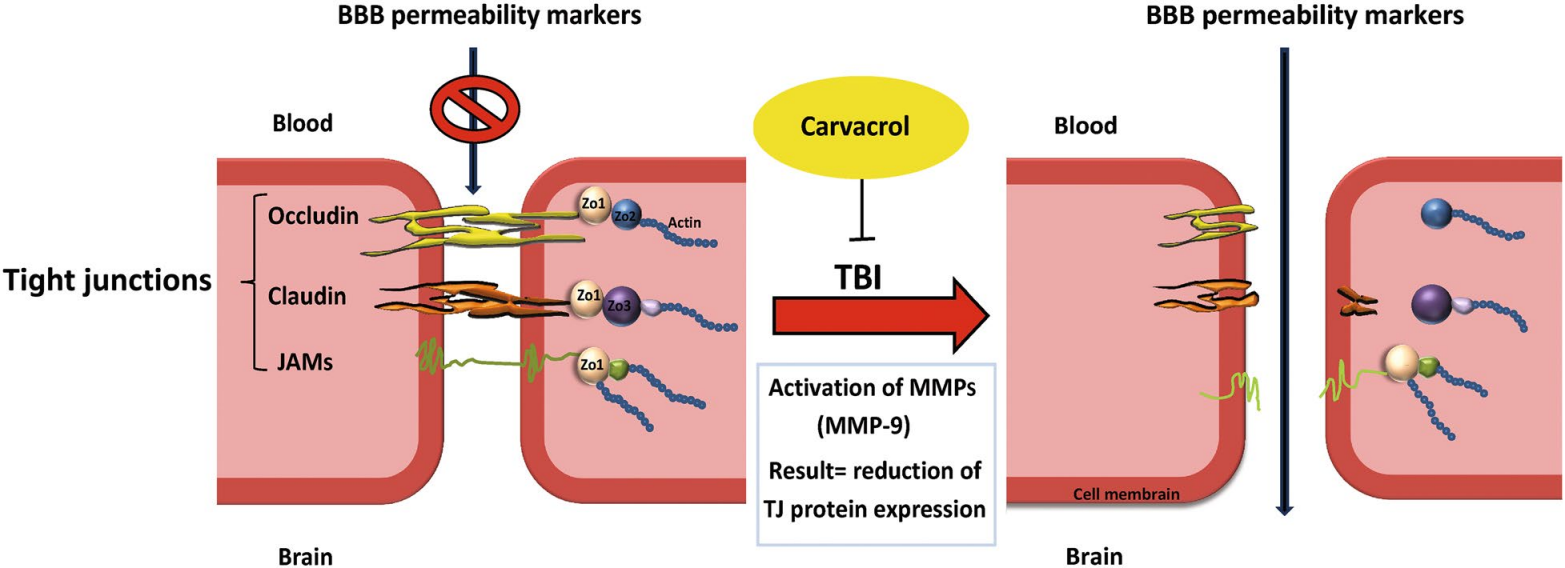
Intraperitoneal administration of Carvacrol 200 mg/kg at 30 min post-TBI suppressed over expression of MMP-9 which mediates degradation of ZO-1, occluding and claudin-5 proteins responsible for increased permeability of BBB. TBI, traumatic brain injury; BBB, blood brain-barrier.

MMP-9 is involved in several cellular processes, such as healing, death, and morphogenesis. After TBI, MMP (including MMP-9) activity is overexpressed, where MMP-9 mediates degradation of ZO-1, occludin, and claudin-5, responsible for increased permeability of the BBB^19^ (Fig. 2). In animal studies and patients with TBI, MMP-9 is implicated in the pathogenesis of cerebral edema and has been effective in predicting the outcomes of neurological disorders^20,21^.

In more severe cases of TBI, cerebral hemorrhage and edema caused by BBB destruction are among the leading causes of mortality in patients with TBI^22^. In TBI, brain edema is divided into cytotoxic and vasogenic edema. Vasogenic edema results from increased BBB permeability allowing extravasation of fluid and plasma into the brain and is associated with acute edema after closed head injury. Cytotoxic edema is cellular swelling typically associated with significant cellular damage and disruption of ion homeostasis. While both forms of edema occur after TBI, vasogenic edema via BBB permeability is best supported in the literature^23^.

Our previous study showed CAR administered 30 min post-TBI significantly decreased brain edema, neuroinflammatory markers, and NF-κB and caspase-3 mediated cell death pathways in rats^24^. In neurotoxic environments, CAR treatment reduces oxidative stress in the brain^7,25,26^; however, the antioxidant properties of CAR have not been evaluated after TBI. We hypothesize that CAR treatment will reduce oxidative stress and MMP-9 activity, thereby preventing the degradation of tight junctions and preserving BBB integrity after experimental TBI, thereby reducing edema. The efficacy of CAR to prevent or attenuate TBI-induced BBB permeability and oxidative stress would be an optimal translatable treatment strategy that could improve TBI outcomes when added to the current standard of care.

## Results

### CAR treatments improved veterinary coma scale (VCS) after TBI

**Figure 3:**
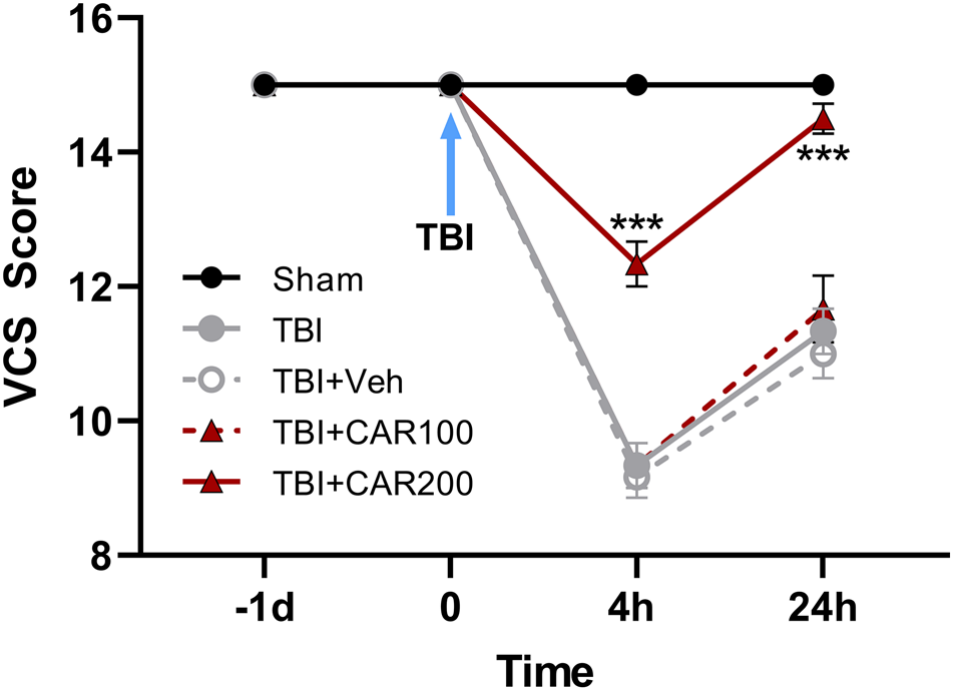
Veterinary coma scale (VCS) scores 4 and 24 h after TBI induction in the different experimental groups (n = 6 in each group). Data are expressed as mean ± SEM. ***P < 0.001 at 4 and 24 h (hr) between TBI + CAR200 and the other groups. TBI decreased the VCS scores significantly at 4 and 24 h in all the groups. However, CAR200 caused greater improvement in the VCS scores compared to the other treatments. TBI, traumatic brain injury; CAR, carvacrol.

Neurological outcomes (VCS scores) among the different groups were evaluated as a function of time post-injury and treatment group (Fig. 3). There was no significant difference in VCS scores between groups before TBI. The VCS scores were significantly decreased in the TBI groups at 4 h (9.33 ± 0.33) and 24 h (11.22 ± 0.23) in comparison with the sham group (15.0 ± 0.0; *P* < 0.001, respectively). However, the VCS scores were increased in the CAR200 group at 4 h (12.33 ± 0.3) and 24 h (14.5 ± 0.22) post-TBI as compared to the TBI + Veh group (*P* < 0.001, respectively). There was no significant difference in the VCS between the TBI, TBI + Veh and TBI + CAR100 groups. VCS scores in the CAR200 naive treated-rats group remained unchanged compared to the sham group (Table 1).

**Table 1:** The effect of CAR200 on naive rats over 24 h.

	Pre injection	30 min	4 h	8 h	24 h
SP (mmHg)	124.75 ± 2.5	117.75 ± 4.1	118 ± 3.4	120 ± 3.8	123 ± 2.6
DP (mmHg)	84 ± 2.7	83 ± 3.7	84 ± 2.6	84 ± 2.9	84 ± 2.2
MAP (mmHg)	98 ± 2.6	94.52 ± 3.8	95 ± 2.6	96 ± 3.1	97 ± 2.3
HR (bpm)	353 ± 6.2	362 ± 10.4	361 ± 10.3	361 ± 28.4	341 ± 24.9
BW(g)	Before	213 ± 8.1	After	212 ± 8.1	
BWC	70 ± 0.24				
VCS	15 ± 0	15 ± 0	15 ± 0	15 ± 0	15 ± 0

No significant change was measured between pre- and post-injection of CAR 200. Data are represented as mean ± SEM. SP, systolic pressure; DP, diastolic pressure; MAP, mean arterial pressure; HR, heart rate; BW, body weight; VCS, veterinary coma scale; BWC, brain water content.

### CAR reduces TBI-induced cerebral edema

**Figure 4:**
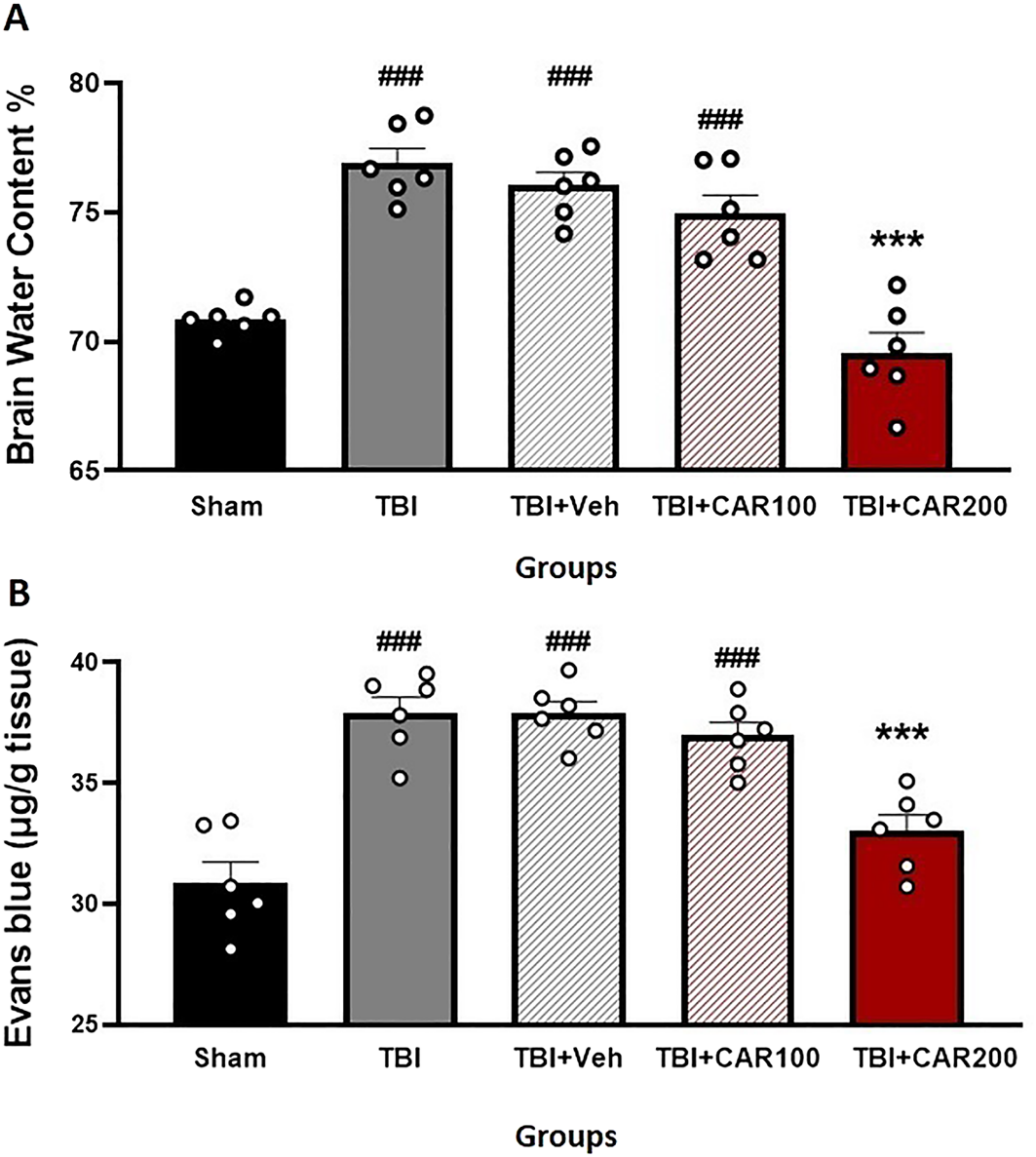
Effect of CAR (200 mg/kg i.p.) on the brain water content (BWC) (**A**) and the Evans blue dye content (μg/g tissue) (**B**) 24 h after TBI induction in the different experimental groups (n = 6 in each group). Data are expressed as mean ± SEM. ^###^*P* < 0.001 represents a significant difference with sham; ***P < 0.01 represents a significant difference with TBI + Veh. TBI, traumatic brain injury; CAR, carvacrol.

Figure 4A shows the effect of different doses of CAR on BWC. The percent BWC in the TBI (76.88 ± 0.58), TBI + Veh (76.02 ± 0.51), and the TBI + CAR100 (74.93 ± 0.73) groups were higher than that of the sham group (70.83 ± 0.23; *P* < 0.001). In contrast, the percent BWC in the CAR200 group (69.55 ± 0.78) was significantly reduced compared to the TBI + Veh group (*P* < 0.001). There was no significant difference in the BWC between the TBI and TBI + Veh groups. CAR200 did not affect BWC in naïve rats compared to the sham group (Table 1).

### CAR decreased blood–brain barrier permeability

To clarify the vasogenic edema, brain EB dye content, a commonly used marker of brain barrier integrity and vascular permeability, was measured (Fig. 4B). The EB dye content in the TBI group was significantly increased compared to the sham group (37.95 ± 0.51 vs. 30.86 ± 0.80, P < 0.001). The TBI + CAR200 group showed a significant decrease in EB dye content compared to the TBI + Veh group (33.01 ± 0.56 vs. 37.88 ± 0.50, P < 0.001), while CAR100 was similar to the TBI + Veh group. The EB dye content was not significantly different between the TBI and TBI + Veh groups. Our data illustrate the significant potency of CAR200 on alleviating vasogenic edema.

### CAR reduced TBI-induced oxidative stress

**Figure 5:**
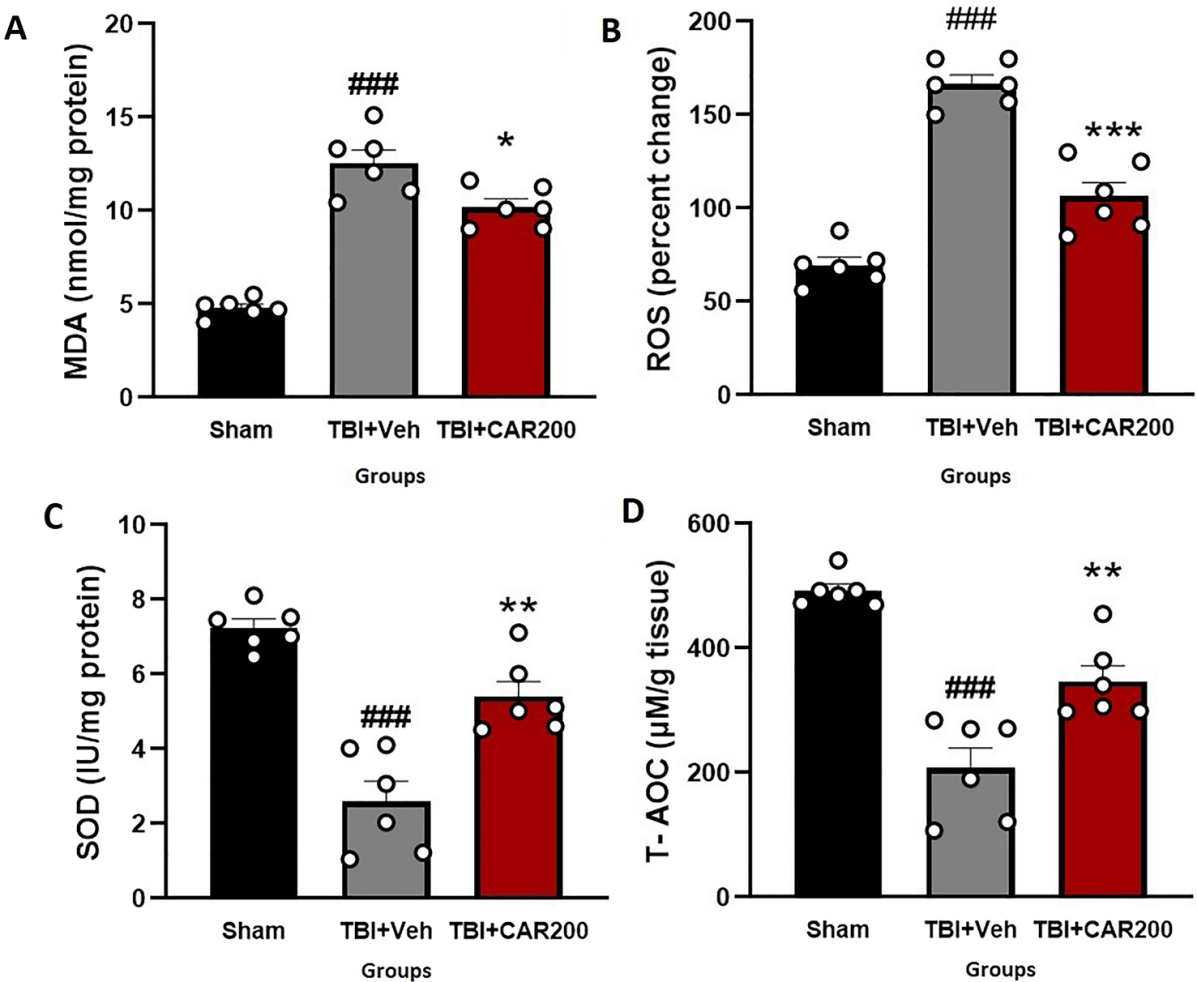
Effect of CAR (200 mg/kg i.p.) on the brain tissue MDA levels (**A**), ROS (**B**), SOD activity (**C**), and T-AOC (**D**) 24 h after TBI induction in the different experimental groups (n = 6 in each group). Data are expressed as mean ± SEM. ^###^*P* < 0.001 represent significant differences with sham; *P < 0.05. **P < 0.01 & ***P < 0.001 represent significant differences with TBI + Veh. TBI, traumatic brain injury; CAR, carvacrol; MDA, malondialdehyde; ROS, reactive oxygen species, SOD, superoxide dismutase; T-AOC, total antioxidative capacity.

The tissue level of MDA increased by 162% in the TBI + Veh group compared to that of the sham group (12.56 ± 0.87 vs. 4.80 ± 0.24, *P* < 0.001). Treatment with CAR200 reduced the MDA levels by 19% compared with the TBI + Veh group (10.19 ± 0.40, *P* < 0.05; Fig. 5A). The level of brain ROS was significantly increased by 58% in the TBI + Veh group as compared to the sham group (166.5 ± 5.95 vs. 69.5 ± 4.17, *P* < 0.001). CAR200 treatment after TBI reduced the level of ROS by 36% compared with the TBI + Veh group (106.75 ± 7.46, *P* < 0.001; Fig. 5B). SOD activity decreased by 66% in the TBI + Veh group compared with the sham group (7.39 ± 0.23 vs. 2.53 ± 0.47, *P* < 0.001). Administration of CAR200 significantly increased SOD activity by 112% compared with the TBI + Veh group (5.36 ± 0.44, *P* < 0.01; Fig. 5C). T-AOC in the TBI + Veh group was decreased by 58% compared to the sham group (207.25 ± 34.70 vs. 492.75 ± 10.28, *P* < 0.001). TBI + CAR200 increased T-AOC by 68% compared to the TBI + Veh group (348.5 ± 25.52, *P* < 0.01; Fig. 5D).

### CAR preserved tight junction proteins after TBI

**Figure 6:**
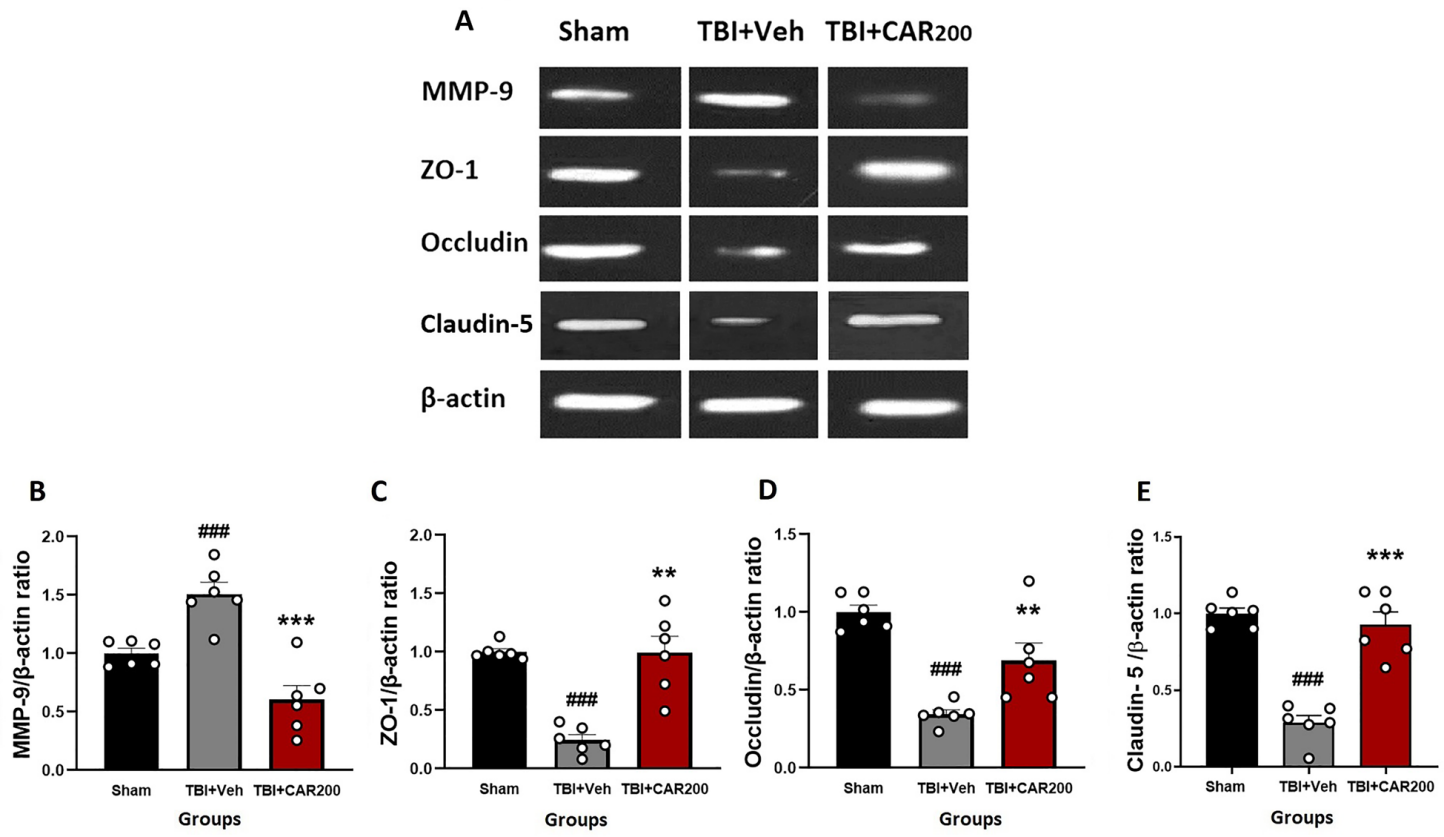
Effect of CAR (200 mg/kg i.p.) on the expression of MMP-9 (**B**), ZO-1 (**C**), occludin (**D**), and claudin-5 (**E**) 24 h after TBI induction in the different experimental groups (n = 6 in each group). Data are expressed as mean ± SEM band density ratio for each group. β-actin was used as an internal control. ^###^*P* < 0.001 represents significant difference with sham; **P < 0.01 & ***P < 0.001 represent significant differences with TBI + Veh. TBI, traumatic brain injury; CAR, carvacrol; MMP-9, matrix metalloproteinase-9, ZO-1, zonula occludens-1. Representative bands of the Western blot from the same run are shown above in (**A**).

The level of ZO-1 (Fig. 6A and C) and occludin (Fig. 6A and D) proteins were significantly reduced by 76% and 70%, respectively, in the TBI + Veh group compared to the sham group (1.0 ± 0.03; 1.0 ± 0.04, respectively) at 24 h after injury (0.24 ± 0.03; 0.30 ± 0.01, respectively; *P* < 0.001). CAR200 treatment significantly increased the expression levels of ZO-1 and occludin 313% and 127%, respectively, compared with the TBI + Veh group (0.99 ± 0.19; 0.68 ± 0.11, respectively). Claudin-5 decreased by 72% in the vehicle-treated injured animals (TBI + Veh group, 0.28 ± 0.05) compared to the sham group (1.0 ± 0.03). CAR200 significantly increased the level of claudin-5 by 229% in comparison with the TBI + Veh group (0.92 ± 0.08; 0.28 ± 0.05, respectively) (Fig. 6A and E).

### CAR reduced MMP-9 protein levels after TBI

A 50% upregulation in MMP-9 level was observed in the TBI + Veh group compared to the sham group 24 h after brain injury (1.50 ± 0.02 vs. 1.0 ± 0.005, P < 0.001) (Fig. 6A and B). CAR200 administration significantly downregulates MMP-9 expression by 60% compared to the vehicle-treated group (0.60 ± 0.15, P < 0.001).

## Discussion

Data support the novel hypothesis that acute treatment with CAR after TBI can attenuate BBB permeability, in part, by decreasing oxidative stress and reducing MMP-9 levels that could be responsible for the preservation of ZO-1, occludin, and claudin-5 levels at 24 h post-injury. Further, the optimal dose of CAR, 200 mg/kg, was identified. Last, the optimal dose of CAR did not cause changes in heart rate, blood pressure, or body weight. Together, these data support that CAR may be beneficial in reducing vasogenic edema as a post-TBI therapeutic intervention that warrants further investigation.

ROS are natural byproducts of oxygen metabolism. Free radicals like superoxide (O_2_^-^), hydroxyl radicals (${\text{OH}}^{ \cdot - }$), and peroxyl radicals (${\text{ROO}}^{ - }$), as well as non-radical species like hydrogen peroxide (H_2_O_2_), are all classified as ROS. The severity of injury in TBI can be estimated by the level of ROS^27^. Superoxide anion is the first to be produced after TBI by cerebral cells by several mechanisms (e.g., arachidonic acid cascade (cyclooxygenase, COX), mitochondrial leakage, activation of microglia, and infiltrating neutrophils and macrophages^12^. Under physiological conditions, the enzyme superoxide dismutase (SOD) can convert superoxide anion into H_2_O_2_ + O_2,_ and glutathione peroxidase then mainly detoxifies H_2_O_2_ into O_2_ + H_2_O and, partly, by catalase and peroxiredoxins^28^. During TBI, each of these neuroprotective systems in the brain can be damaged, eventually promoting oxidative cell damage^29^. Nitric oxide is a substantial signaling gaseous molecule found in the rat nervous, immune, and cardiovascular systems, and whose homeostasis is greatly affected by TBI^30,31^. NO radical and superoxide anion (NO + ${\text{O}}_{{2}}^{ \cdot - }$) generates peroxynitrite (${\text{ONOO}}^{ \cdot - }$), which degenerates into various unstable reactive nitrogen species (RNS). Peroxynitrite, RNS, and ROS actively participate in lipid peroxidation, DNA damage, and protein oxidation^32^. By definition, lipid peroxidation is a free radical-mediated event, that can result in the breakdown of polyunsaturated fatty acids in lipid membranes, producing MDA^33^ leading to oxidative neural injury post-TBI.

Our results showed that the ROS and MDA levels were significantly higher in the TBI + Veh group compared to the sham group, similar to other groups using this experimental model of TBI^34,35^.Our data indicated that the percentage of ROS and MDA generation was lower in the CAR-treated group (36% and 19%; respectively) than in the vehicle-treated group, indicative of the neuroprotective role of CAR treatment. There is a growing body of evidence on the role of CAR on free radical scavenging, which has been also previously shown in in vitro and in vivo studies^36^. For instance, Samarghandian and colleagues administrated CAR to rats that were exposed to chronic stress. They showed that CAR not only alleviates free radicals such as peroxide, H_2_O_2_, superoxide, and NO but also improves the activity of antioxidant enzymes, such as SOD, CAT, and GPx. In their study, CAR exerted a protective effect on the hippocampus of rats via decreasing lipid peroxidation^7^. In addition, Wang et al.^37^ revealed that CAR alleviated lipid peroxidation in the hippocampus of ethanol-exposed rats. In line with the antioxidant effects of CAR, Li et al.^38^ have shown that CAR inhibits the oxidative response in a rat model of focal cerebral ischemia by increasing SOD activity and decreasing MDA levels. Therefore, probably part of the protective actions of CAR in our study may be related to its increasing antioxidant power (SOD & T-AOC) and reducing oxidative stress. Clinical studies also indicate the antioxidant effects of CAR^39^. For example, Khazdair and colleagues recently administered CAR for two months on 20 patients exposed to sulfur mustard (SM)-inducing lung disorders 27–30 years ago. CAR significantly reduced MDA, increased the SOD and CAT factors, and improved forced vital capacity and peak expiratory flow in these patients^40^.

Our data also showed that CAR200 treatment reduced TBI-induced injury severity by over 50% at 4 h post-injury, returning to sham levels by 24 h post-injury, demonstrating that improved oxidative homeostasis and preserved BBB could promote behavioral recovery. It is well known that the prevention of lipid peroxidation is vital to improving neurological outcomes in clinical and preclinical studies so that the improved VCS scores in the CAR-treated group may relate to a limited level of MDA^41^. Furthermore, we speculate that the improvement of neurological behavior in the group treated with CAR is due to the correlation of antioxidant property of this substance and apoptotic process, because excessive oxidative stress induces resulting in releasing cytochrome c, a protein that plays a crucial role in cell death and is inhibited by Bcl-2 protein, from outer mitochondria membrane (OMM). Then, cytochrome c forms the apoptosome complex in the cytosol. These two mechanisms converge at the level of effector caspases such as caspase-3 and caspase-7, resulting in cleavage of cellular proteins and apoptosis^42^. It has also been demonstrated that decreasing caspase-3 activation and apoptotic cell death promotes functional recovery in animals following TBI^43,44^. It is important to note that 24 h after administration of CAR200, in our previous study, Bax/Bcl-2 protein ratio and cleaved caspase-3 synthesis in the brains of rats decreased, and consequently neurological scores improved^24^. Therefore, CAR may have reduced neurological deficit post-TBI by reducing ROS and subsequent reduction in apoptosis.

Of interest, several studies have found a potent link between oxidative stress and MMP-9 in the pathophysiology of BBB damage after TBI^18^. Multiple cellular and molecular mechanisms that induce MMP-9 activation can be triggered by oxidative stress^45^. MMP-9 contributes to the degradation of tight junction proteins (ZO-1, occluding and claudin-5) affecting BBB permeability^46^. While ZO-1 and occludin are important regulators of the blood–brain barrier, claudin-5 plays a crucial role in maintaining barrier integrity, where claudin-5 levels have been linked to increased permeability and disruption of the blood–brain barrier^47^. Our findings indicate that CAR effects on BBB permeability and brain edema might be attributed to a reduction in oxidative stress resulting in the modulation of MMP-9 expression and BBB tight junction proteins. Supporting these outcomes, in-vitro studies showed the capability of CAR to decrease the MMP-9, MMP-2, and ROS in human glioblastoma and human mesenchymal stromal^48,49^.

One of the possible mechanisms through which CAR reduced MMP-9 could be c-Jun N-terminal protein kinase (JNK). According to recent research by Lu et al.^17^, the oxidative stress and JNK pathway significantly increase the magnitude of MMP-9 activation resulting in the degradation of tight junction proteins and increased BBB permeability in the rat brains after TBI. Gholijani et al.^50^ suggested that CAR can inhibit JNK pathways in lipopolysaccharide (LPS)-stimulated mouse macrophages. The underlying mechanisms through which CAR administration changes JNK expression might differ in various animals and cell types and has not been evaluated after TBI.

In addition to affecting the JNK pathway, ROS also has stimulating effects on the transcription of NF-κB which appear to play a major role in perpetuating the immune response to TBI and is known to increase genetic transcription of pro-inflammatory mediators such as TNFa and IL-6^51^. It has been clarified that inflammatory cells and BBB permeability are related to neurological defects following TBI^52^. In this case, inhibition of NF-κB blocks MMP-9 upregulation in ischemic brain endothelium^53^. It has been also observed that there is a correlation between neurological scores and reducing MMP-9 mRNA and protein expression levels in patients with brain trauma^21^. Based on our previous work, we showed that CAR-treated animals had significantly lower pro-inflammatory cytokines and NF-κB protein post-TBI^24^. Thus, it is reasonable to speculate that better neuronal function following CAR treatment may be associated with BBB protection and suppression of the ROS/NF-κB/MMP-9 pathway.

Limitations. (1) Evans blue used in our study is an inexpensive and widely available transcellular marker of albumin extravasation across the blood–brain barrier, often used to determine vasogenic edema and BBB permeability after TBI. Due to the increased chance of detection in controls, it is less precise than paracellular tracer permeability, like dextran and inulin^54^. However, in a study by Liao et al.^55^, EB and dextran were compared and indicated that EB could detect BBB permeability, but non-specific signals were reduced in shams using dextrans. So, the increase in extravasation in injured animals in these experiments was likely due to BBB permeability caused by the TBI. (2) The experiments only used male rats, despite growing evidence indicating there are sex differences in the acute glial response, including astrocytes, microglia, and infiltrating macrophages that can lower the amount of MMP activation and subsequent degradation of the BBB at the 24 h and 7 days time points. However, there is support that this may be due to a delayed response after TBI, where more chronic time points reveal similar long-term pathology^56,57^. Based on these publications, it is likely that a sex-dependent response would be present that requires females to be included in future experiments and in the evaluation of long-term outcomes. (3) Our experiments only evaluated outcome measures at 24 h. Longitudinal evaluation of both pathophysiological sequelae and the influence on long-term neurological function would improve overall interpretation regarding any potential benefit or early intervention with CAR. (4) Our findings demonstrated specific antioxidant properties of CAR; however, CAR may also have broader neuroprotective effects beyond its antioxidant properties. For example, CAR has been shown to have anti-inflammatory effects, contributing to activated microglia and astrocytes, where reducing inflammation can help to maintain BBB integrity^58^. It is also possible that CAR may reduce inflammation, which in turn reduces oxidative stress^59,60^. Additionally, CAR may regulate other pathways that contribute to BBB integrity and neuroprotection, indicating the potential for also mediating aspects of cytotoxic edema, which is typically more associated with focal injuries. Further research is needed to fully understand the potential therapeutic applications of CAR in this context.

## Conclusions

Our findings indicate that CAR prevents the loss of ZO-1, occludin, and claudin-5 proteins after TBI, through a MMP-9 signaling pathway. Our results also revealed that the impact of CAR treatment on MMP-9 might be partially attributed to a decrease in the generation of oxidants such as MDA and ROS and an increase in antioxidants like SOD activity and T-AOC. The 200 mg/kg dose of CAR did not cause any acute changes in blood pressure, heart rate, or body mass, indicating that it may be the optimal dose to evaluate the long-term impact of CAR on pathophysiological processes. Thus far, data support CAR as an affordable, accessible (over-the-counter) essential oil that may be valuable in countering acute effects of TBI.

## Materials and methods

### Chemicals preparation

Tween 20 and CAR were purchased from Merck Millipore (Darmstadt, Germany) and Sigma-Aldrich (282197, Germany).

### Animals and study design

**Figure 7:**
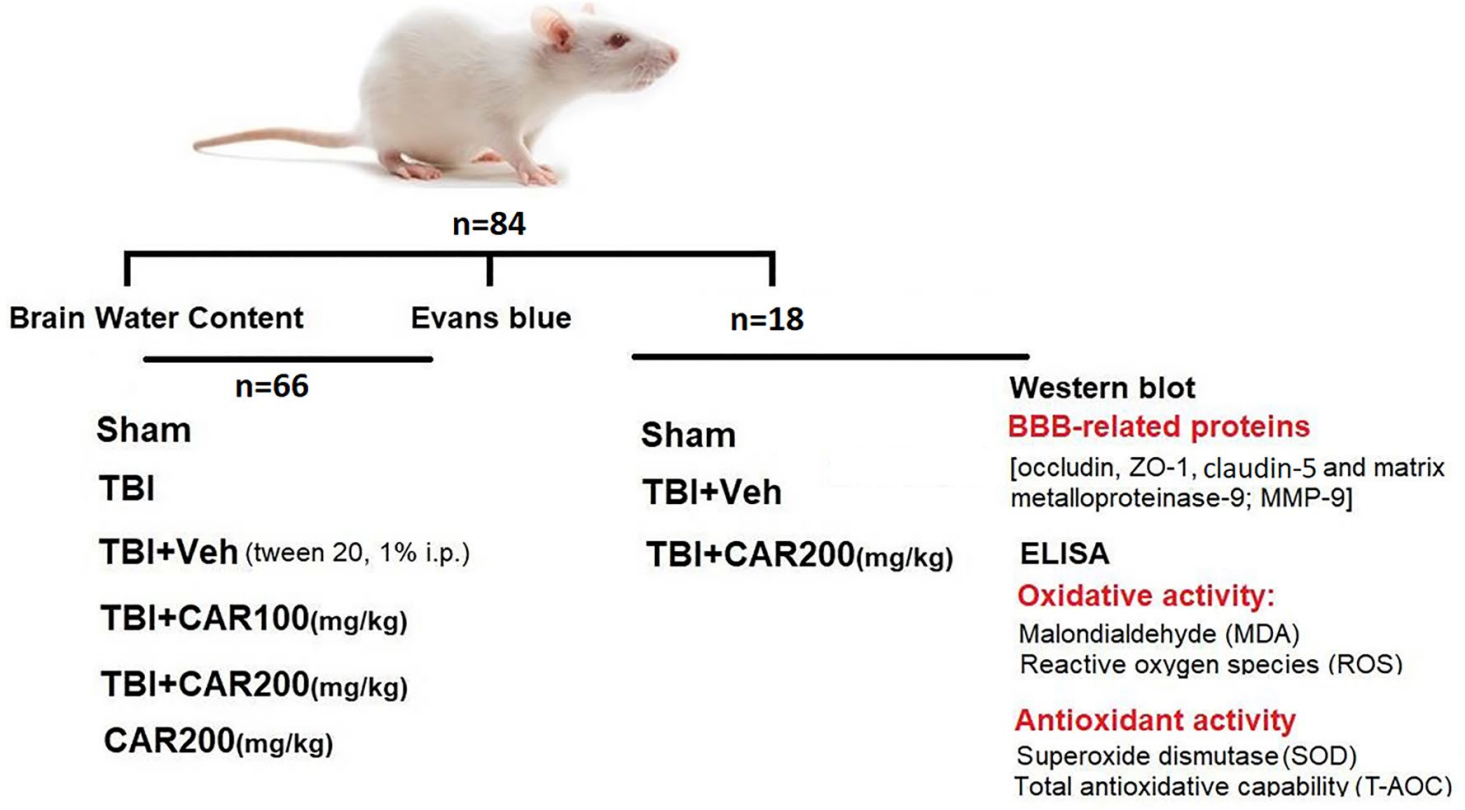
Experimental design including animal treatment and the different cohorts included.

Male Wistar rats (200–250  g) were kept in an air-conditioned room (Temp: 22–25 °C) with standard 12  h of light/dark cycles and free access to food and water. Animals were randomly divided into six groups as described (Fig. 7):

1. Sham group: these rats were not exposed to brain trauma.
2. TBI group: these rats were exposed to the brain trauma but did not receive treatment.
3. TBI + Veh group: these rats were intraperitoneally injected with vehicle (tween 20, 1% i.p.)^11,23^.
4. TBI + CAR100 group: these rats were intraperitoneally injected with CAR (100 mg/kg, i.p).
5. TBI + CAR200 group: these rats were intraperitoneally injected with CAR (200 mg/kg, i.p).
6. CAR200 group: these naive rats were intraperitoneally injected with CAR (200 mg/kg, i.p).

All treatments and vehicles were administered 30 min post-TBI induction. To achieve 90% power- to detect statistical significance at a 95% confidence interval, 6 rats were assigned to each of the groups.

A dose of CAR100 was ineffective in the injured treated-animals, whereas CAR200 significantly decreased BWC, EB and improved VCS (Figs. 3 and 4). In this regard, our previous dose–response analysis revealed that CAR (250 mg/kg) caused drowsiness (somnolent-like behavior)^24^. As a result, higher doses were avoided due to the risk of interfering with neurological consciousness-dependent results (e.g., VCS).

To evaluate the possible effect of CAR200 on naive rats, the general health such as blood pressure and heart rate were measured along with body weight, brain water content and VCS in this animals. No significant change was observed compared to the sham group (Table 1). As the result we did not involved CAR-treated control in the rest of study. Furthermore, TBI and TBI + Veh groups showed no significant difference when tween 20 (1% w/v) was administered as a vehicle; thus, we only investigated the experimental parameters in the TBI + Veh group. As a result, the number of utilized animals were reduced.

The rats were anesthetized and sacrificed with sodium thiopental [80 mg/kg, i.p]^61^ 24 h after the TB. All experiments were performed in accordance with relevant guidelines and regulations and adhere to the ARRIVE guidelines (https://arriveguidelines.org/) for the reporting of animal experiments. The study reviewed and approved by the local ethical committee of the Kerman University of Medical Sciences (Ethics code No. IR.KMU.REC.1398.351).

### Diffuse traumatic brain injury (TBI) induction

Before the TBI induction, all rats were intubated. The TBI method employed was moderate diffuse brain injury, according to the Marmarou methodology^62,63^. Briefly, animals were anesthetized with Ketamine (50 mg/kg) and Xylazine (5 mg/kg) i.p; then, a metal disc with dimensions of 10 mm (diameter) and 3 mm (thickness) was placed and affixed to the animal skull. A 300 g weight was dropped onto the anesthetized rat head from a height of 2 m. Post-injury, rats were immediately connected to the breathing pump (TSA animal respiratory compact, Germany). The intra-tracheal tube was withdrawn when spontaneous breathing was restored, and rats were placed in individual cages. The site of brain damage was visible on H and E-stained sections (supplementary data 1).

### Brain water content (BWC) assessment

To quantify cerebral edema, the brain water content (BWC) of each rat was evaluated 24 h after TBI induction. Brains were quickly removed from anesthetized rats and weighed wet-dry to determine percent water content. After the wet weight was recorded, the brains were incubated at 100 °C (Memmert, Germany) for 24 h, followed by recording the dry weight. The percent water content of each brain sample was assessed using the following formula^62,64^:

$$Brain\,\;water\;\,content\;\,\left( \% \right)\;\, = \;\, \left[ {\left( {wet\,\;weight\;-\;dry\,\;weight}\right)/wet\;\, weight}\right]\, \; \times \;\,\left. {100} \right]$$

### Determination of blood–brain barrier permeability

Four hours after the TBI, Evans blue (EB) (20 mL/kg) was injected intravenously, according to O'Connor’s instructions^62^. Rats were anesthetized, and using saline infusion EB dye was washed for one hour. The brains of the animals were then removed and homogenized in phosphate-buffered saline (PBS). Trichloroacetic acid was used to precipitate the protein, then cooled and centrifuged (at 2000 cycles/min for 10 min). The supernatant was utilized to evaluate the EB absorbance at 610 nm using a spectrophotometer (UV/VIS, Spectrometer, UK). More dye in the brain tissue indicates greater BBB permeability^62^. The EB dye content was calculated using the following formula:

$$Evans\,\;blue\;\,dye\;\,\left( {\mu g} \right)\;\, in\;\,brain\;\,tissue\,\;\left( g \right)\,\; = \;\, \left({13.24\,\;\times \;\,20\;\,\times\;\,absorbance} \right)/tissue\,\;weight$$

### Measurement of mean arterial pressure (MAP)

Systolic and diastolic blood pressure was recorded by the tail-cuff technique using an NIBP ML125 system (ADInstruments, Australia.blood pressure was measured by placing animals’ tail in the cuff of a monometer*. MAP* = *DP* + *1/3(SP−DP)*^65^*.*

### Spectrophotometric assessment of MDA and SOD

Brains were removed from the skull and hemisected into two hemispheres. The left cerebral hemispheres of rats were homogenized in ice-cold PBS following TBI. The samples were then centrifuged at 2000 g for 10 min at 4 °C. The supernatant was utilized to measure malondialdehyde (MDA) level and SOD activity according to available spectrophotometric kits (ZellBio Antioxidant GmbH, Germany)^66^.

### Total antioxidant capacity assessment

Benzie and Strain Method was used to evaluate total antioxidant capacity (TAC)^67^. In brief, a working solution of ferric reducing antioxidant power (FRAP) was prepared to contain 25 mL of 0.3 M acetate buffer (pH 3.6), 2.5 mL of 10 m MTPTZ solution, and 2.5 mL of 20 mM FeCl3. A total of 1.5 mL of the FRAP reagent was added to 50 μL of homogenized brain tissue and left for 10 min at 37 °C. The absorbance was read at 593 nm as a measurement for the TAC. The calibration curve was plotted with a concentration of 50–1000 FeSO_4_ in a FRAP reagent. Data were recorded as μM/mg of tissue.

### Determination of reactive oxygen species (ROS)

ROS quantitation in brain tissue homogenates was measured using the 2′,7′-dichlorofluorescein diacetate (DCFH-DA) assay, as illustrated previously^68^. Briefly, the homogenate was diluted at 1:20 (v/v) with PBS buffer, pH 7.4. The reaction mixture (200 μL) containing 190 μL of homogenate and 10 μL of 1 mM DCFH-DA was incubated for 30 min at 37 °C. The conversion of DCFH-DA to the fluorescent product 2′,7′-dichlorofluorescein was recorded using a microplate reader (Perkin Elmer Victor 2) at an excitation/emission wavelength of 484/530 nm. The ROS level is represented as a percentage of the sham group as being set at 100%.

### Western blot analysis of MMP-9, ZO-1, Occludin and Claudin-5 expression

A tissue homogenizer (Hielscher UP200, Germany) was used to homogenize the right hemispheres of the brain in 700 μL ice-cold RIPA lysis buffer (Sigma; R0278), 1 mM protease inhibitors (Sigma; P2714-IBTL), and 1 mM sodium orthovanadate (13721-39-6, Sigma-Aldrich). The protein homogenate was centrifuged for 15 min at 4 °C at 14,000 rpm. Protein concentration was assessed using the Bradford method (Bio-Rad Laboratories, München, Germany). Equal amounts of protein (40 g) were electrophoretically separated on a 10% SDS-PAGE gel and transferred to nitrocellulose membranes (Hybond ECL, GE Healthcare Bio-Sciences Corp., NJ, USA). The membranes were blocked with 5% non-fat milk for 2  h before overnight incubation at 4 °C with ZO-1 (1:200, R40.76: sc-33725), Occludin (1:200, F-11: sc-133255), Claudin-5 (1:200, clone EPR7583, ab131259), and MMP-9 (1:200, E-11: sc-393859). After three times washing with TBST (15 min each), the membranes were incubated for 2 h at room temperature with the secondary antibodies anti-mouse (1:1000, m-IgGκBP-HRP: sc-516102), anti-rat (goat polyclonal, ab97057), and goat anti-rabbit (1:1000 ab205718). All antibodies were diluted in a blocking buffer (TBST and 5% non-fat dried milk). β-actin immunoblotting was used to control the loading (C4: sc-47778). Antibody-antigen complex signals were amplified via the Enhanced Chemiluminescence (ECL) method and then detected using the Gel Documentation System (Bio-Rad). Lab Works software was used to determine the density of the bands. Finally, the membranes were placed for half an hour at 55 °C in the stripping solution, rinsed with TBST, and blocked again for β-actin (antibody from Cell Signaling Technology Inc., Beverly, MA, USA; 1:200)^69,70^. Protein levels were normalized to β-actin and then normalized to sham.

### Statistical analysis

Statistical analysis was performed using the SPSS, version 17.0 (SPSS, Inc., Chicago). The normality of data distribution was evaluated using the Shapiro–Wilk test. In addition, One-way ANOVA was used to examine experimental data, BWC, EB, ELISA, and Western blot, followed by an HSD or Tukey test for post-hoc analysis. Repeated measure ANOVA was done to compare VCS data between groups at different times. The paired samples t-test, is used to assess the change in MAP data. Data are represented as the mean ± SEM. *P* < 0.05 was considered statistically significant as shown in the figures.

### Supplementary Information

Supplementary Information 1.

Supplementary Information 2.

## Data Availability

Data for this work is archived and publicly available upon request to the corresponding author.
