# Supplementary figures and images for: Carvacrol decreases blood–brain barrier permeability post-diffuse traumatic brain injury in rats
Source: Sci Rep. 2023 Sep 4;13:14546. doi: 10.1038/s41598-023-40915-x (PMC10477335; doi:10.1038/s41598-023-40915-x)

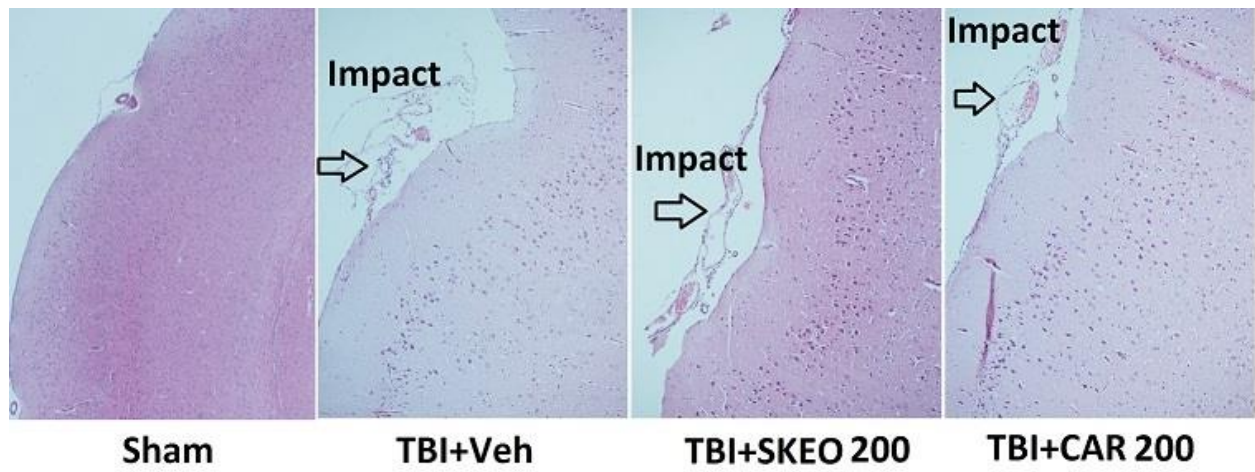

The site of brain damage in H and E-stained sections

Supplement: Supplementary file 1 — Supplementary Information 1. [file 41598_2023_40915_MOESM1_ESM.pdf]
