# Supplementary material for: Carvacrol decreases blood–brain barrier permeability post-diffuse traumatic brain injury in rats
Source: Sci Rep. 2023 Sep 4;13:14546. doi: 10.1038/s41598-023-40915-x (PMC10477335; doi:10.1038/s41598-023-40915-x)
Supplement: Supplementary file 2 — Supplementary Information 2. [file 41598_2023_40915_MOESM2_ESM.pdf]

Western Blot full gels of ZO-1 (A), Occludin (B) , MMP-9 (C), and Claudin-5 (E) Expression in Control=Sham ,TBI+Veh and TBI+Carvacrol 200mg/kg(CAR). β-actin immunoblotting was used to control the loading (F&D).The yellow rectangulars show used bonds in the the main manuscript (Fig 6A).

A

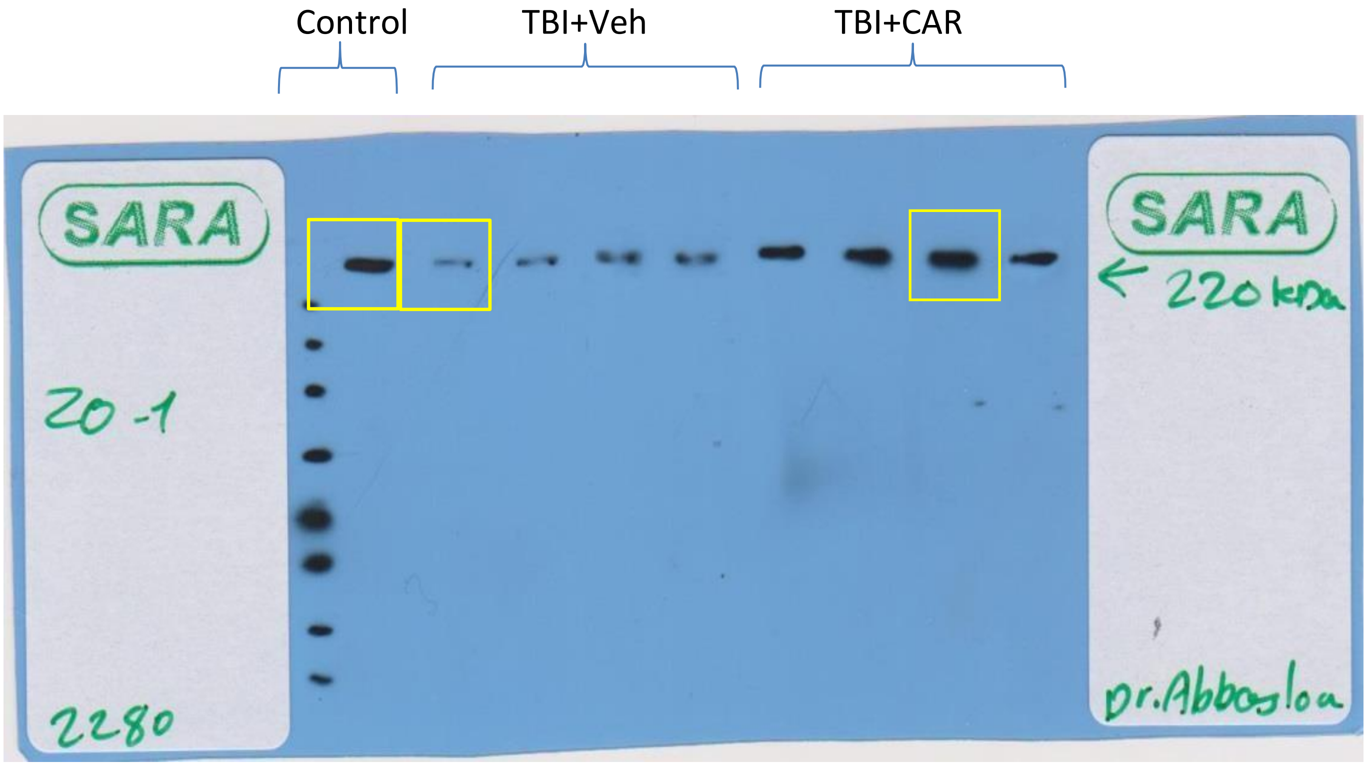

B

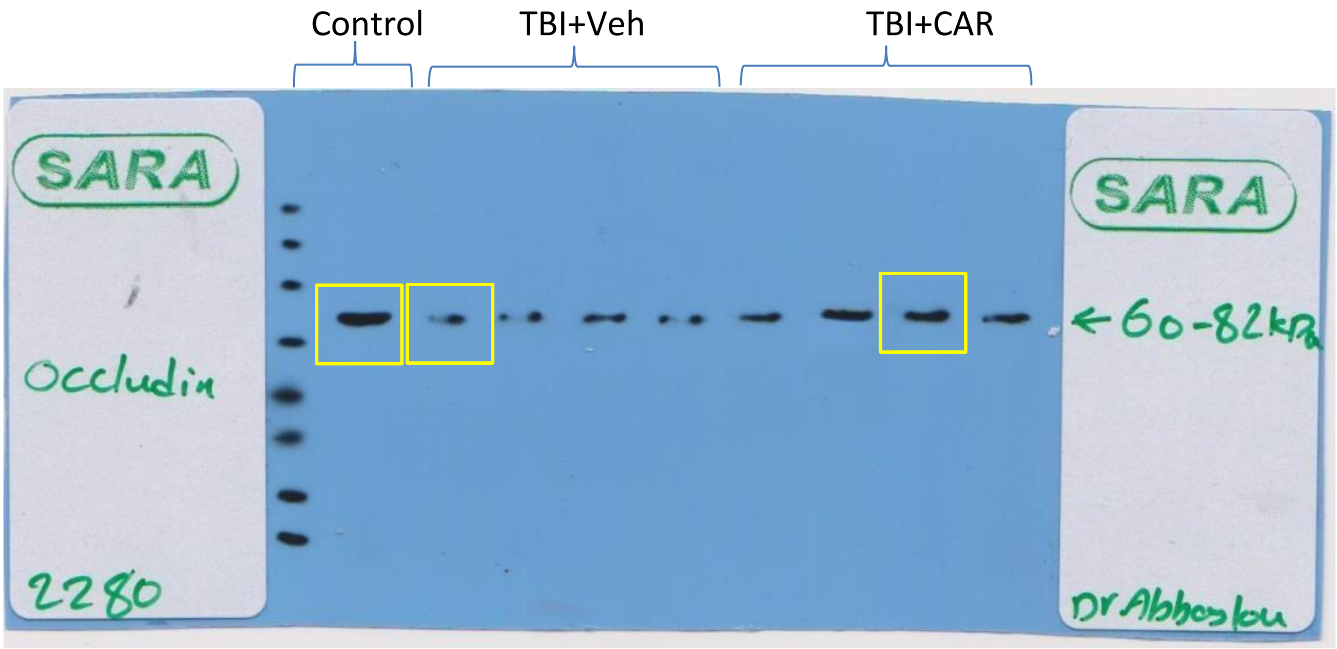

C

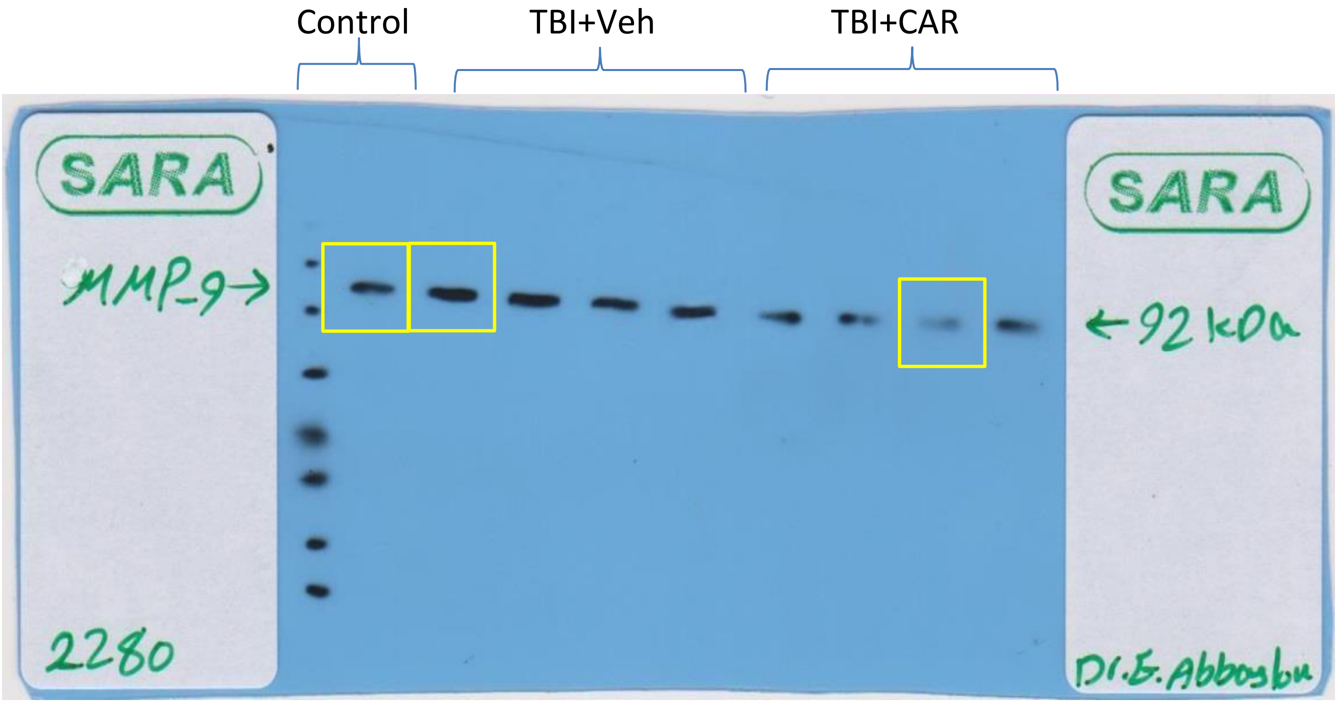
Control
TBI+Veh
TBI+CAR
SARA
MMP-9→
SARA
←92 kDa
2280

D

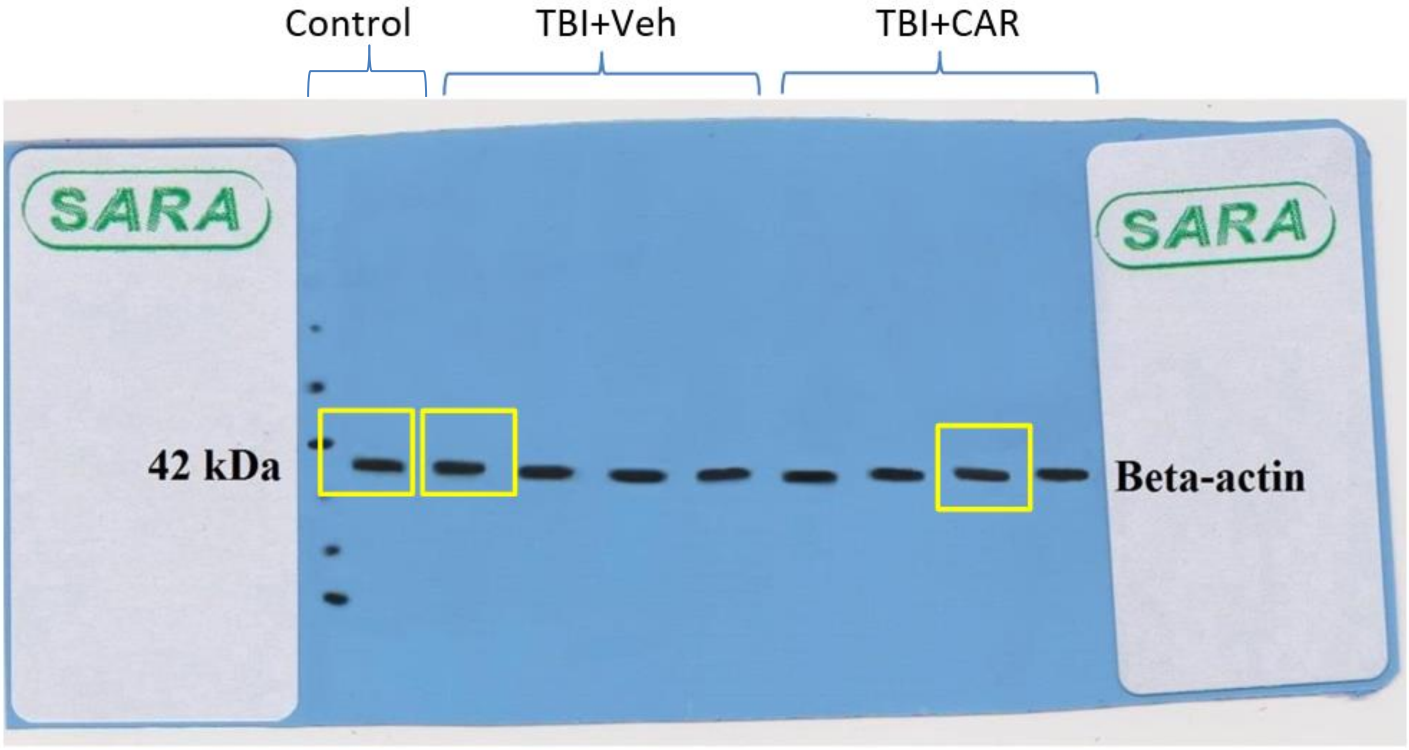
Control
TBI+Veh
TBI+CAR
SARA
SARA
42 kDa
Beta-actin

E

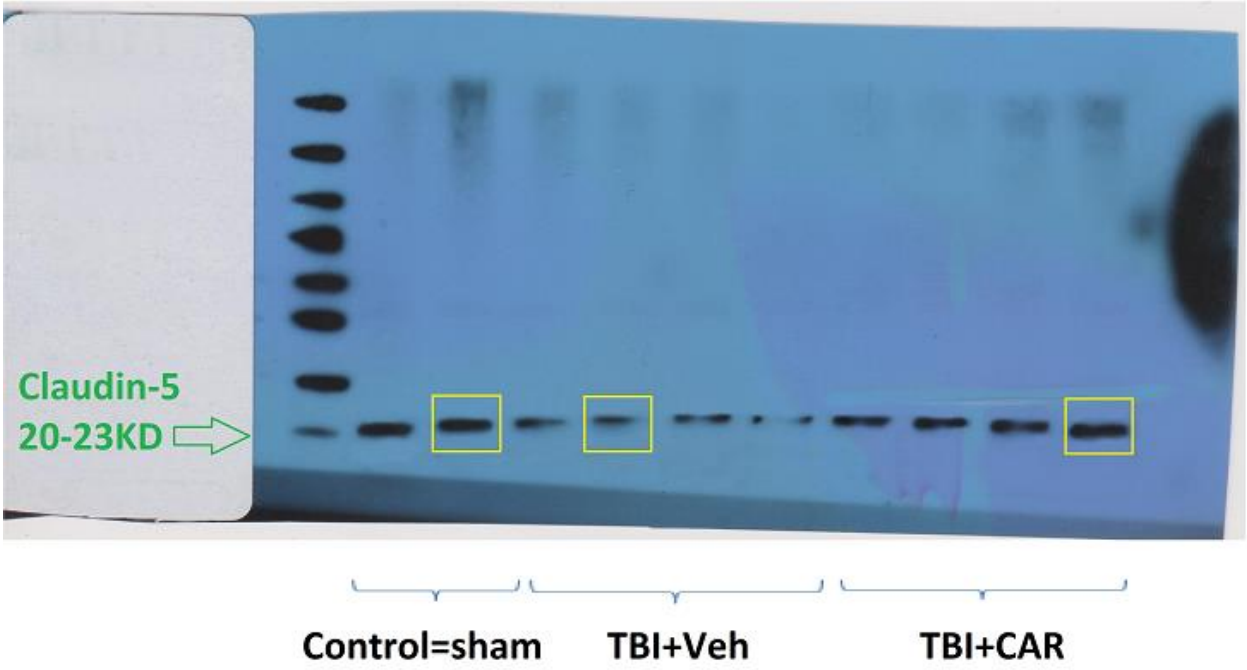
Claudin-5
20-23KD
Control=sham
TBI+Veh
TBI+CAR

F

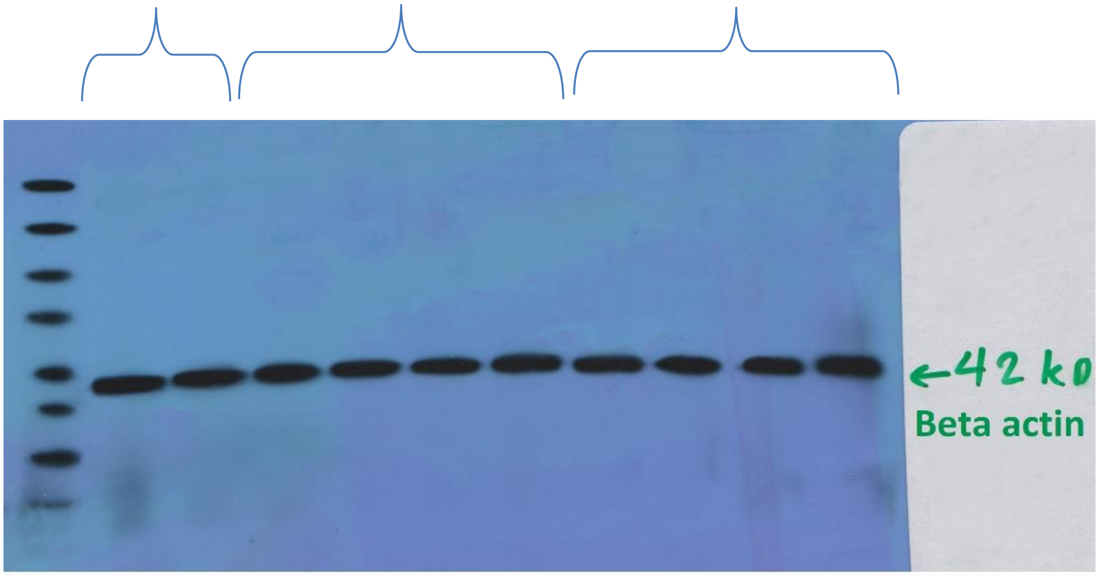
←42 kD
Beta actin
